# Depression and burnout among Chinese nurses during COVID-19 pandemic: a mediation and moderation analysis model among frontline nurses and nonfrontline nurses caring for COVID-19 patients

**DOI:** 10.1186/s12888-023-05006-1

**Published:** 2023-08-31

**Authors:** Jingjun Wang, Xia Huang, Mengmeng Wang, Lei Huang, Ya Wang

**Affiliations:** 1 Department of Nursing, West China Hospital, Sichuan University/West China School of Nursing, Sichuan University, Chengdu, China; 2 Mental Health Center, West China Hospital, Sichuan University/West China School of Nursing, Sichuan University, Chengdu, China; 3 School of Nursing, Peking University, Beijing, China; 4 School of Nursing, Tongji Medical College, Huazhong University of Science and Tecnology, Wuhan, China

**Keywords:** Burnout, Depression, Coping styles, Interpersonal relationship, COVID-19, Nurses

## Abstract

**Background:**

During the COVID-19 pandemic, nurses' workload increased dramatically, and nurses faced the risk of infection and multiple ethical dilemmas. In such a situation, nurse burnout was elevated, which tended to exacerbate depression in nurses. Although previous studies have demonstrated the relationship between burnout and depression among nurses, the exact mechanisms remain unclear. Furthermore, environmental factors are also essential to a person's psychological health. Therefore, this study intended to investigate the potential mechanisms of depression caused by nurse burnout and whether burnout among frontline nurses during the COVID-19 pandemic exacerbated its effect on depression in nurses as an environmental factor.

**Methods:**

A total of 4517 nurses were enrolled in this study. A moderated mediation model was established to investigate the relationship between burnout and positive coping styles, interpersonal relationships, and depression utilizing the SPSS PROCESS 3.3 macro. The direct effect of burnout on depression was also investigated with the moderated mediation model.

**Results:**

The indirect effects of positive coping styles (β = 0.04, 95% CI: 0.03 to 0.04) and interpersonal relationships (β = 0.12, 95% CI: 0.10 to 0.13) were revealed. Being a frontline nurse caring for COVID-19 patients moderated the direct effect of burnout on depression (β = 0.12, 95% CI: 0.08 to 0.16).

**Conclusion:**

This study offers strong evidence for the mediating role of positive coping styles and interpersonal relationships in the relationship between nurse burnout and depression, in addition to illustrating the need for more psychological support for frontline nurses caring for COVID-19 patients.

## Background

Since COVID-19 first emerged in Wuhan, China, in December 2019, increased fear and death have occurred worldwide [1]. According to the reports of the COVID-19 Weekly Epidemiological Update, which was launched on 23 November 2022 by the WHO, 0.634 billion infections and 6.6 million deaths have been reported [2]. Nurses, the main defenders of public health during the COVID-19 pandemic, were exposed to an increasing number of infections and experienced a heavy workload for prolonged periods of time, causing depression [3–5]. For instance, a study that focused on medical staff confronted with COVID-19 in China indicated that 54% of nurses suffered from depression [3]. Furthermore, compared to other medical staff, nurses had a more severe depression [3]. Since nurses’ psychological conditions are closely related to the quality of care and recovery of patients, it is necessary to address depression in nurses [6].

Depression among nurses has been proven to be associated with burnout, which is a syndrome that contains three components, emotional exhaustion, depersonalization, and decreased personal accomplishment, and originates from prolonged job pressure [7–9]. In terms of the definition of burnout and the symptoms of depression, a strong similarity was demonstrated between burnout and depression [10]. The empirical overlap of burnout and depression is supported by much of the literature [11–13]. Leiter and Durup et al. argued that the content of emotional exhaustion, one of the core components of burnout, is a typical symptom of depression [14]. Another core component of burnout, depersonalization, also has similarities to depression, namely, social withdrawal, which is a common characteristic of depersonalization and depression. The components of low self-efficacy and perceived helplessness involved in depression also share characteristics with the burnout dimension of decreased personal accomplishment [14]. Previous studies indicated that burnout was a risk factor for depressive disorders among nurses [10]. Nurses who reported a clinical level of burnout were 43 times more likely to suffer from major depressive disorder [15]. Thus, nurses’ depression might be escalated by high-level burnout. Previous studies have also suggested that the optimal approach to preventing depression in nurses is to reduce work stress and burnout [16, 17].

However, most of the stressors, such as heavy workloads and complicated clinical situations, are difficult to eliminate in the real world [17]. According to the stress and response patterns proposed by Lazarus, individuals’ responses to central pressure can be affected by their coping styles, social support, and cognitive evaluation [18]. Coping styles have been indicated as a critical stress mediator according to stress and response patterns [18]. Research prior to COVID-19 identified that nurses’ stress reactions can be mitigated by utilizing adaptive coping styles [19]. For instance, Garcia-Arroyo indicated that coping styles are directly associated with burnout, and decreased positive coping is associated with decreased personal accomplishment [20]. Furthermore, coping styles can directly impact the consequences of burnout, such as depression [21]. Shao et al. stated that coping strategies may play a critical role in the maintenance or exacerbation of depression and anxiety, and positive coping was negatively associated with depression [22]. Thus, the relationship between burnout and depression might be mediated by positive coping styles.

Apart from coping styles, effective interpersonal relationships appears to be closely related with burnout [23]. During the pandemic, nurses endured intense workloads for long periods of time, which may have resulted in nurses not devoting enough energy to address interpersonal relationships [24]. Poor interpersonal relationships are closely related with depression and have proven to be a risk factor for depression [25, 26]. Studies of medical students suggested students’ mental health, academic performance, and interpersonal relationships could be affected by academic-related burnout and cause depression and poor psychosomatic states [27]. According to the stress and coping pattern, positive interpersonal relationships may partially play a social support role in the relationships between stress-mediating variables and, in this way, influence the relationship between burnout and depression. Therefore, burnout might have interacted with depression in nurses during the pandemic through individuals’ coping styles and interpersonal relationships.

Furthermore, frontline nurses caring for COVID-19 patients, i.e., those who directly worked with COVID-19-infected patients, experienced higher levels of work pressure than nonfrontline nurses, i.e., those who worked with uninfected patients [28–30]. An individual’s environment also affects their psychological state [31]. An individual's context can also act as an important situational variable [32]. During the COVID-19 pandemic, nurses who took care of COVID-19-infected patients were more vulnerable to burnout [28]. Mohammad et al. compared the pressure among nurses during and before the COVID-19 pandemic in Iran and concluded that the level of work stress among nurses during the pandemic was significantly higher than that before the pandemic [33]. In addition, since previous studies have indicated that depression can be caused by burnout, the impact of burnout on depression might be enhanced. A study conducted in Iran indicated that the depression level among nurses was significantly higher during the COVID-19 pandemic than before the pandemic [33]. Caring for COVID-19 patients could be a moderator affecting nurses’ psychological status, and the relationship between burnout and depression among nurses might be moderated by whether they are frontline nurses caring for COVID-19 patients.

In summary, the relationship between burnout and depression might be mediated by nurses’ coping styles and interpersonal relationships and might be moderated by whether they are frontline nurses caring for COVID-19 patients. However, most of the studies focused on the relationship between burnout and depression among nurses have investigated the direct effect of burnout on depression rather than the internal mechanisms of this effect [33, 34]. The potential mediators or moderators were also not considered. There is no clear explanation regarding how burnout affected the risk factors for depression among nurses during the pandemic [35].

Therefore, this study aimed to explore the relationship between burnout and positive coping styles, interpersonal relationships and depression among nurses during the pandemic, and further explore whether the relationship between burnout and depression was exacerbated by being a frontline nurse for COVID-19 patients to provide more evidence to alleviate depression among nurses.

**Fig. 1:**
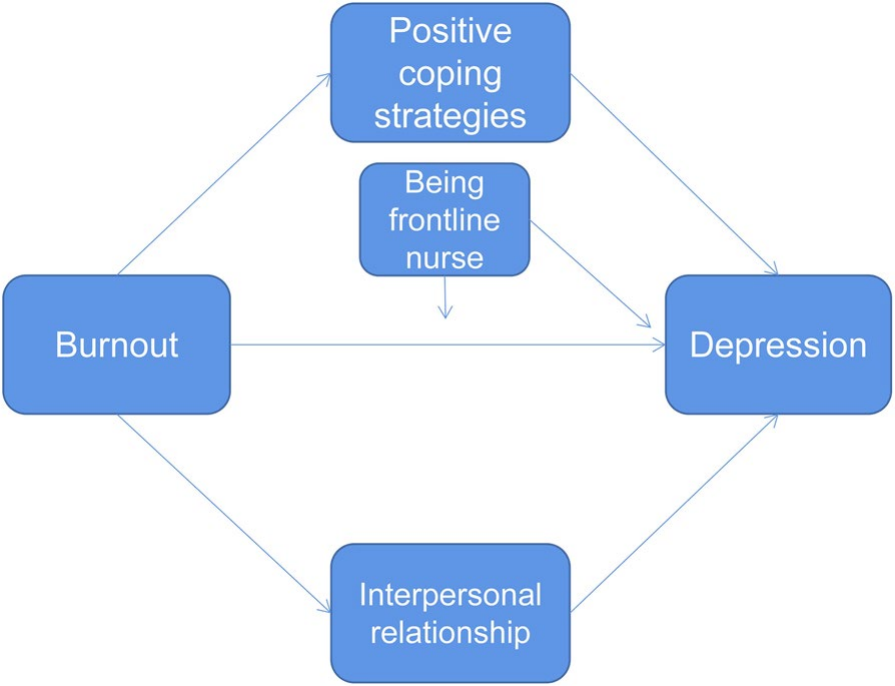
Concept model of this study

This study utilized the stress and coping pattern proposed by Lazarus et al., which states that when an individual is stimulated by a stressor, the stress response that emerges is influenced by multiple stress-mediating variables such as coping style, social support, and cognitive appraisal [18]. Based on the available literature and the purpose of this study, the stress and coping pattern proposed by Lazarus et al. was utilized as the theoretical basis of the present study. Burnout was considered as major stressor, positive coping strategies as well as interpersonal relationships were considered as stress mediators, and depression among nurses was considered as a stress response. Furthermore, the effect of environmental factors on the relationship between nurse burnout and depression was also considered. The following hypotheses were formulated in this study:

- H1: The positive coping styles of nurses play a mediating role between burnout and depression.
- H2: The nurses’ interpersonal relationships play a mediating role between burnout and depression.
- H3: The direct effect of burnout on depression was greater for frontline nurses than for nonfrontline nurses during the COVID-19 pandemic.
  The conceptual model of this study is shown in Fig. 1.

## Methods

### Sample and settings

A large sample online survey was conducted. The researchers selected the hospitals to be studied based on the following criteria: (1) The selected hospitals were public hospitals, and the scale of the hospital was a tertiary hospital or above (the number of hospital beds was 500 or above); community hospitals, private hospitals, and clinics that provide primary medical, preventive, rehabilitative, and health care services were not be included in this study; (2) Comprehensive hospitals were selected to ensure the representativeness of the study; specialty hospitals (excluding specialty COVID-19 hospitals) were not be included in this study; (3) In the case of specialty COVID-19 hospitals, the hospital scale was 100 hospital beds or above; and (4) Hospitals for which the manager could be successfully contacted and who agreed for the research to be conducted in their hospital. Ultimately, a total of ten hospitals in Shanghai, Chongqing, Sichuan, Gansu, and Yunnan (including five specialized COVID-19 hospitals) met the above requirements, and the managers were willing to participate in this study. Departments of specialized COVID-19 hospitals, such as fever outpatient clinics, isolation wards, and observation wards, were enrolled in this study. Five researchers connected the managers of all of the departments online by using the software WeChat or Tenchent conference within the selected hospitals. Inclusion criteria were as follows: (1) more than 18 years old; (2) no cognitive impairment or psychiatric disorders; and (3) registered nurses. In the demographic information section of the questionnaire, the study subjects were asked to provide personal information related to the inclusion exclusion criteria, and the researcher screened the study subjects based on the information they filled in, with the subjects who did not meet the inclusion criteria being removed.

### Investigation procedures

Prior to formal data collection, an online questionnaire was created by the researchers utilizing the WJX software (www.wjx.com), which contained three components: a note about the purpose of the study, the methodology, and considerations for participation; an informed consent form; and the questionnaire. After obtaining consent from the manager of each department, the online questionnaires were sent to them. After the online questionnaires were sent to the managers of each department, the department manager organized a half-hour online meeting at a time when most of the nurses were off (e.g., Friday evening), and the researcher explained the basic content of the survey to the nurses. After the explanation, the questionnaires were sent to each nurse by the managers of the departments, and each nurse was free to choose the time to complete the questionnaires within one week according to their own situation. A small number of nurses on duty conducted additional meetings based on departmental scheduling. They were also asked to complete the questionnaires within a week.

Nurses had to read the note for at least 30 s, then the informed consent form for at least 30 s and select the option of willing to participate in this study. Then, they were able to complete and submit the questionnaires. A total of 6103 questionnaires were sent from March 2022 to September 2022, and 5500 nurses chose to participate in this study. Of these, 5350 nurses met the inclusion criteria.

**Fig. 2:**
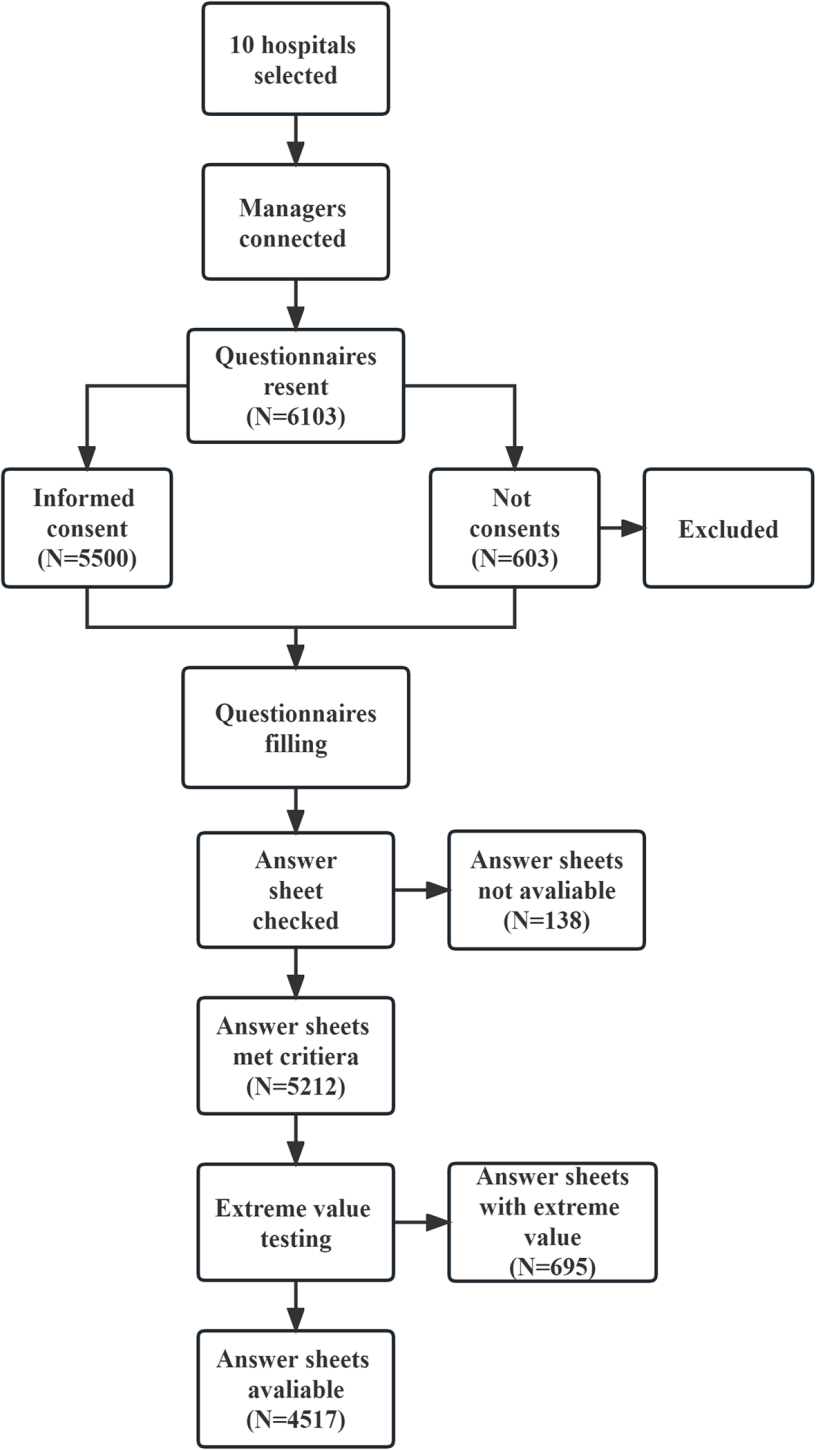
The process of sampling

Strict quality control measures were utilized to ensure the authenticity of the questionnaire data in this study. For example, a person could only fill out a questionnaire after reading and signing the informed consent. Each questionnaire was filtered using automatic filtering rules and manually inspected by the researcher after submission. The filter criteria were as follows: (1) Questionnaires with similar questions with opposite answers were excluded; (2) Questionnaires for which the same response was recorded for each item were excluded; and (3) Questionnaires that took less than one minute to complete were also be excluded. Questionnaires could not be submitted unless they were complete. After extreme value testing and data cleaning, a total of 4517 study subjects were included. The details of the sampling process are demonstrated in Fig. 2.

## Measures

### Demographic characteristics questionnaires

A demographic characteristics questionnaire was developed to collect the nurses’ general information, including gender, age, level of hospitals, educational background, monthly income, job title, actual positions, marital status, and fertility status.

### Depression

The depression subscale of the Depression-Anxiety-Stress Scale-21 (DASS-21) was utilized. The DASS was developed by Lovibond et al. in 1995, with the initial objective of distinguishing and defining common emotional disorders, such as depression, anxiety, and stress, through a consistent measurement system for administration and scoring [36]. The scale can be applied as a psychometric aid for clinical diagnosis and as a rapid and effective subject screening tool for relevant studies [36, 37]. The DASS-21 is a revised and streamlined version of the DASS that enhances the efficiency of identifying and assessing symptoms of corresponding emotional disorders, while retaining the same stable factor structure and equally favorable reliability and validity as the full version of the DASS [36]. The depression subscale of DASS-21 has seven items measured on a four-point Likert scale, with higher scores representing more severe depression. The Cronbach's α coefficient of this study is 0.854. The total Kaiser–Meyer–Olkin measure of this study was 0.898. Results of confirmatory factor analysis indicated the χ^2^ /DF of the subscale is 2.392, GFI = 0.999, RMSEA = 0.016, indicated that this scale has good construct validity. The total score was obtained by multiplying the sum of all subscale scores by 2. Depression was classified as normal for a score of 0 to 9, mild for a score of 10 to 13, moderate for a score of 14 to 20, and severe for a score of 21 or higher [36].

### Burnout

The Maslach Burnout Inventory-General Survey (MBI-GS) developed by Schaufeli et al., which contains the three dimensions emotional exhaustion, depersonalization, and decreased personal accomplishment and 16 items, was utilized [38]. The Chinese version of the MBI-GS was revised by Chaoping Li et al. and contains 15 items. The Chinese version of the MBI-GS is measured on a 7-point Likert scale, with 0 representing "never" and 6 representing "very often" [39]. The Cronbach's α coefficients for emotional exhaustion, depersonalization, and decreased personal accomplishment were 0.918, 0.883, and 0.890, respectively. The total Cronbach's α coefficient of the MBI-GS was 0.942. The Kaiser–Meyer–Olkin measures for emotional exhaustion, depersonalization, and decreased personal accomplishment were 0.877, 0.788, and 0.901, respectively. The total Kaiser–Meyer–Olkin measure of the MBI-GS was 0.952. Results of confirmatory factor analysis indicated the χ^2^/DF of the subscale is 3.483, GFI = 0.965, RMSEA = 0.023, indicated that this scale has good construct validity.

### Interpersonal relationships

The Comprehensive Interpersonal Relationship Diagnostic Scale developed by Richang Deng was utilized. It is used to measure individuals' psychological distress in interpersonal relationships, with 28 items in 4 dimensions: conversational behavior, communicative distress, treating others with respect, and heterosexual interaction. Each item is answered yes (1 point) or no (0 points). The higher the total score is, the more serious the interpersonal distress [40]. The Cronbach's α coefficient of this study was 0.878. The Kaiser–Meyer–Olkin measure of the Comprehensive Interpersonal Relationship Diagnostic Scale was 0.930. Poor interpersonal relationships were classified as mild for a score of 0–9, moderate for a score of 10–14, and severe for a score of 15 or above [40]. Results of confirmatory factor analysis indicated the χ^2^ /DF of the subscale is 5.490, GFI = 0.930, RMSEA = 0.064.

### Positive coping styles

The Simplified Coping Style Questionnaire (SCSQ) developed by Yaning Xie was utilized in this study [41]. The questionnaire has 20 items, with questions 1 to 12 measuring positive coping styles and questions 13 to 20 measuring negative coping styles. Only the positive coping style subscale was used in this study. SCSQ was based on a 4-point Likert scale, A score of 0 indicates no adoption and a score of 3 indicates frequent adoption. The Cronbach's α coefficient of this study is 0.913. The Kaiser–Meyer–Olkin measure of the Simplified Coping Style Questionnaire (SCSQ) is 0.942. Results of confirmatory factor analysis indicated the χ^2^/DF of the subscale is 4.586, GFI = 0.977, RMSEA = 0.233, indicated that this scale has good construct validity.

### Statistical analysis

Data were analyzed by the software SPSS 26.0 and the SPSS PROCESS 3.3 macro. Frequency and percentage statistics were adopted to describe the demographic characteristics of nurses. Quantitative data was described by mean ± SD. The normality of the data was tested and a nonnormal distribution was detected for all variables. Therefore, the Spearman correlation analysis and nonparametric tests were conducted to investigate the potential relation between variables. Before testing the moderated mediation model, the mediation role of positive coping styles and interpersonal relationship were tested by model 4 in PROCESS 3.3 macro. Then, the moderated model was tested by model 5. A bootstrap method with 10,000 samples was conducted to reduce bias, and 95% confidence intervals were tested. If 0 was not included in the 95% confidence intervals, then the path coefficient was regarded as significant. Since the moderating variables are dichotomous, they are directly enrolled in the model processing.

## Results

### Demographic features of the subject

A total of 4517 subjects were enrolled in this study. The majority of the participants were women (4377, 96.9%). More than half of the participants (2942, 65.1%) had obtained a bachelor’s degree. The majority of nurses in this study were salaried at less than ¥10,000: 1762 (39.0%) of the nurses earned less than ¥5000 per month and 2606 (57.7%) earned ¥5000–10, 000 per month. The majority of the participants (3218, 71.2%) were married, and nearly half of the participants (2040, 45.2%) had a child. A total of 2334 (51.7%) were frontline nurses caring for COVID-19 patients, and 3671 (81.3%) were afraid of being infected with COVID-19. Details of the participants’ demographic features are demonstrated in Table 1.

**Table 1:** Socio-demographic characteristics of participants

Variables	N/%	H(Z)	*P*
**Gender**		-3.58	< 0.001^*^
Men	140 (3.1)		
Women	4377 (96.9)		
**Age**		15.25	0.002^*^
18–29	1956(43.3)		
30–39	1737(38.5)		
40–49	604(13.4)		
> = 50	216(4.8)		
**Educational background**		28.69	< 0.001^*^
Junior high school and below	7(0.2)		
Senior high school	10(0.2)		
Secondary school graduation	100(2.2)		
College degree	1438(31.8)		
Bachelor degree	2942(65.1)		
Master degree and above	20(0.4)		
**Monthly income**		7.616	0.107
< 5000	1762(39.0)		
5000–10,000	2606(57.7)		
10,000–15,000	124(2.7)		
15,000–20,000	19(0.4)		
> 20,000	6(0.1)		
**Marital status**		10.480	0.015^*^
Unmarried	1162(25.7)		
Married	3218(71.2)		
Divorces	126(2.8)		
Others	11(0.2)		
**Fertility status**		4.035	0.26
Childless	1548(34.3)		
1 child	2040(45.2)		
2 children	912(20.2)		
3 children or above	17(0.4)		
**Positions**		1.176	0.56
Department head nurses	83(1.8)		
Head nurses	199(4.4)		
Nursing team leaders	360(8.0)		
Nurses	3875(85.8)		
**Professional titles**		9.86	0.02^*^
Junior nurses	2885(63.9)		
Intermediate nurses	1309(29.0)		
Associate Senior	291(6.4)		
Senior nurses	32(0.7)		
**Whether fear of COVID-19 infection**		-2.28	0.023^a^
Yes	3671(81.3%)		
No	846(18.7%)		
**Whether frontline-nurses of COVID-19 patients**		-25.174	< 0.001^*^
Yes	2334(51.7%)		
No	2183(48.3%)		

^*^indicated *P* < 0.05

### Comparison of depression levels among nurses with different demographic characteristics

Significant connections between nurses’ gender, age, educational background, marital status, professional titles and the depression levels were detected by nonparametric tests. Fear of COVID-19 infections and being frontline nurses caring for COVID-19 patients were likely to be related with nurses’ depression. Details are shown in Table 1.

### Correlations between major variables

The results of Spearman correlation analysis indicated that burnout among nurses was closely related with nurses’ depression (*r* = 0.681, *p* < 0.001). Positive coping styles were negatively related with nurses’ depression (*r* = -0.360, *p* < 0.001). Poor interpersonal relationships were positive associated with nurses’ depression (*r* = 0.551, *p* < 0.001). Furthermore, burnout, positive coping styles, and interpersonal relationships were related with each other (Table 2).

**Table 2:** Descriptive statistics and related analysis results of major variables

Variables	Mean	Severity	(N, %)	1	2	3	4
**1.Positive coping strategy**	22.8 ± 7.47			1			
**2.Interpersonal relationship**	5.09 ± 4.85	Mild	3421 (75.7)	-0.287^**^	1		
		Moderate	850 (18.8)				
		Severe and above	246 (5.4%)				
**3.Depression**	6.00 ± 6.06	No depression	3165 (70.1)	-0.360^**^	0.551^**^	1	
		Mild	577 (12.8)				
		Moderate	695 (15.4)				
		Severe and above	80 (1.8)				
**4.Burnout**	6.71 ± 3.61			-0.341^**^	0.491^**^	0.681^**^	1

^**^indicated *P* < 0.01

### Mediation analysis by parallel mediation analysis

Mediating effects analysis was conducted after controlling for demographic variables. The results of mediation analysis of positive coping styles and interpersonal relationships are demonstrated in Table 3. The total effect of burnout on depression was estimated as β = 0.66 (95% CI: 0.64 to 0.68). Direct effect of burnout on depression was estimated as β = 0.51 (95% CI: 0.48 to 0.53). The total indirect effect of burnout on depression was also estimated (β = 0.15, 95% CI: 0.14 to 0.17). Furthermore, the mediation analysis indicated that positive coping styles mediate the relationship between burnout and depression (β = 0.03, 95% CI: 0.03 to 0.04). Interpersonal relationships also mediated the relationship between burnout and depression (β = 0.12, 95% CI: 0.11 to 0.13). Details of the mediation analysis are shown in Table 3.

**Table 3:** Mediation effect of interpersonal relationship and positive coping strategy (by parallel mediation analysis)

Variables	Total effect			Direct Effect			Indirect Effect		
	**B**	**LLCI**	**ULCI**	**B**	**LLCI**	**ULCI**	**B**	**LLCI**	**ULCI**
**Burnout**	0.66	0.64	0.68	0.51	0.48	0.53	0.15	0.14	0.17
**Positive coping strategy(M1)**							0.03	0.03	0.04
**Interpersonal relationship(M2)**							0.12	0.11	0.13
**Difference of indirect effect between M1 and M2**							-0.09	-0.10	-0.07

M1 indicated positive coping strategies, M2 indicated interpersonal relationship

### Moderated mediation analysis

After parallel mediation analysis was performed, a moderated mediation analysis was conducted. On the basis of the consideration of conditioning factors, the total indirect effect of burnout on depression was estimated as β = 0.15 (95% CI: 0.14 to 0.17). Positive coping styles mediated the relationship between burnout and depression (β = 0.04, 95%CI: 0.03 to 0.04). Interpersonal relationships also mediated the relationship between burnout and depression (β = 0.12, 95% CI: 0.10 to 0.13).

**Fig. 3:**
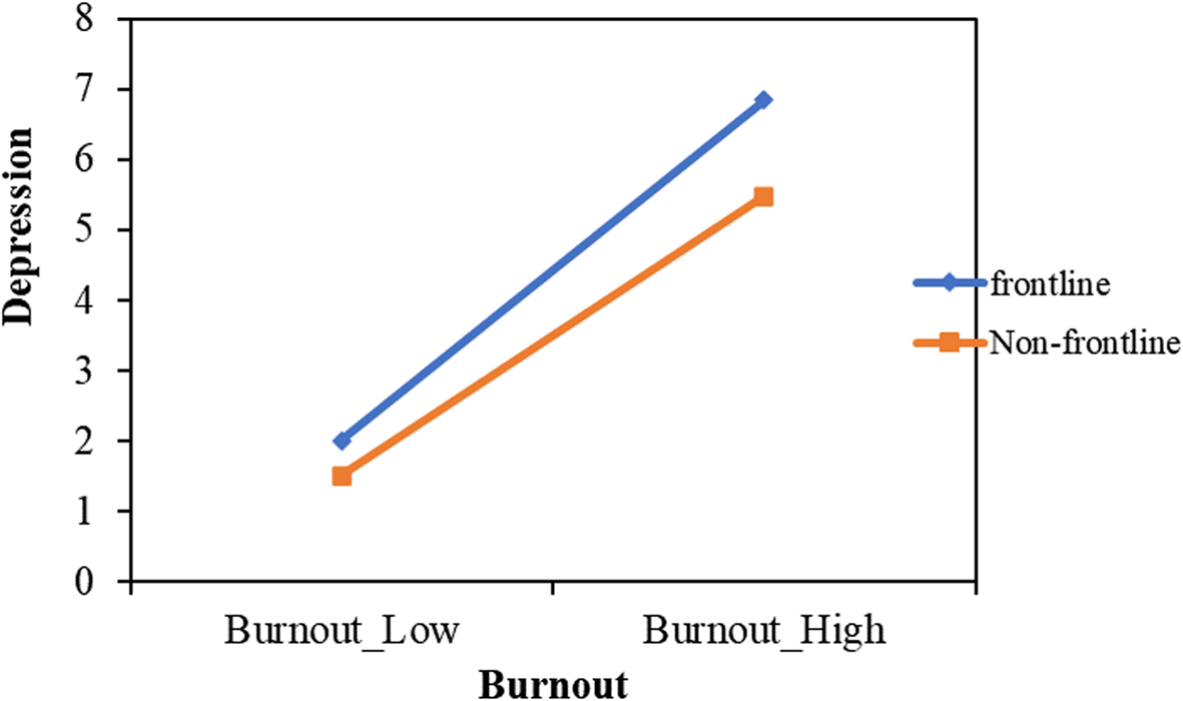
The conditional effect of burnout on depression

**Fig. 4:**
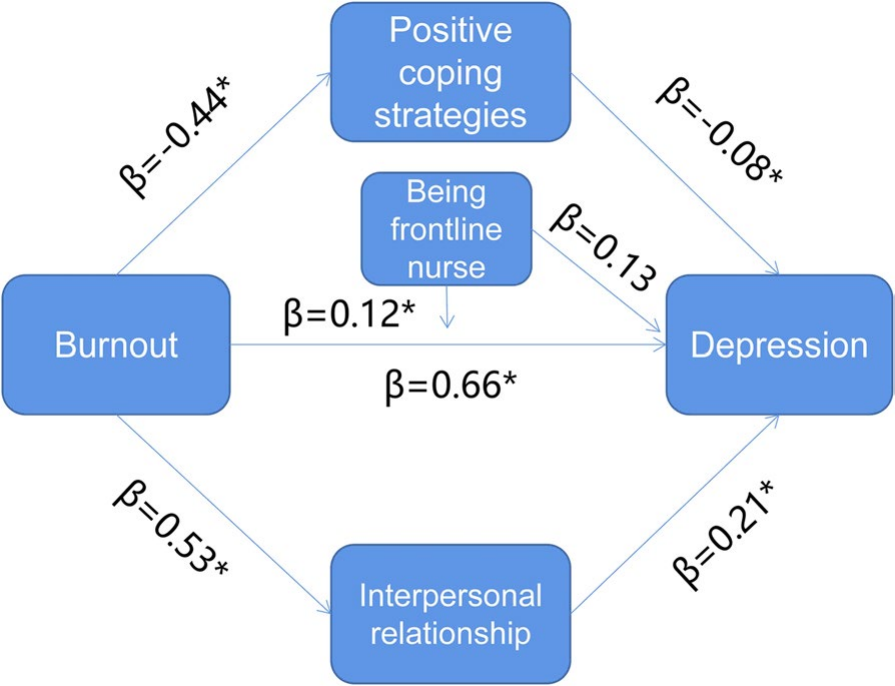
Moderated mediation model of this study

Moderated analysis identified that being frontline nurses for COVID-19 patients moderated the direct effect of burnout on depression (β = 0.12, 95% CI: 0.08 to 0.16). Details shown in Table 4. Regarding the direct impact of burnout on depression, frontline nurses (β = 0.54, 95% CI: 0.51 to 0.57) and nonfrontline nurses (β = 0.42, 95% CI: 0.38 to 0.45) were both significant (Fig. 3) (Table 5). The final moderated mediation model was demonstrated in Fig. 4.

**Table 4:** Moderated mediation analysis results for the relationship between burnout and current depression

Variables	M1			M2			Depression		
	**B**	**LLCI**	**ULCI**	**B**	**LLCI**	**ULCI**	**B**	**LLCI**	**ULCI**
**Burnout**	-0.44^*^	-0.48	-0.41	0.53^*^	0.50	0.56	0.66^*^	0.59	0.73
**Positive coping strategy(M1)**							-0.08^*^	-0.10	-0.06
**Interpersonal relationship(M2)**							0.21^*^	0.19	0.23
**W**							0.13^*^	0.10	0.17
Burnout^*^W							0.12^*^	0.08	0.16
**Total indirect effect**							0.15	0.14	0.17
**Indirect effect(M1)**							0.04	0.03	0.04
**Indirect effect(M2)**							0.12	0.10	0.13
**F**	R^2^ = 0.13 F = 109.47			R^2^ = 0.23 F = 229.18			R^2^ = 0.52 F = 494.31		

M1 indicated positive coping strategies, M2 indicated interpersonal relationship

^*^indicated that *P* < 0.05, W indicated that whether being a frontline-nurse for COVID-19

**Table 5:** Conditional indirect effects of burnout on depression

Variables	X → Depression		
	**B**	**LLCI**	**ULCI**
**Frontline nurses**	0.53^*^	0.51	0.57
**Non-frontline nurses**	0.42^*^	0.38	0.45

X indicated burnout

^*^indicated that *P* < 0.05

## Discussion

This study investigated the potential mechanisms of burnout and its impact on depression in nurses. This study verified the hypothesis that positive coping styles and interpersonal relationships played a mediating role between burnout and depression. Burnout could lead to depression among nurses by impairing effective coping styles as well as interpersonal relationships. Compared to nurses who were not frontline nurses, frontline nurses caring for COVID-19 patients were more likely to experience a magnified impact of burnout on depression.

### Mediating role of positive coping styles and interpersonal relationships

Concordant with hypothesis 1, positive coping styles could mediate the association between burnout and depression, which was similar to the views pointed out by previous studies [42–44]. For instance, Wang et al. indicated that coping styles could mediate the relationship between perceived pressures and mental distress (anxiety, depression) among physicians in China [42]. Zhou et al. also indicated that a positive coping style can serve as a protective factor to mediate the relationship between burnout and anxiety. Physicians with lower levels of burnout are more likely to address issues through positive coping styles, which in turn lead to a reduction in psychological distress [44]. According to conservation of resource theory (COR), both the loss of existing resources and the failure to acquire new resources can trigger a stress response in individuals [45]. When confronted with a desperate situation of resource depletion, the individual's self-protective defense mechanism will be triggered, and the individual will redeploy coping styles [45]. When the individual's burnout is severe and psychological resources are depleted, the individual may adopt less positive coping styles and more negative coping to protect themselves due to defense mechanisms [46]. Individuals are likely to experience depression when their positive coping style is reduced and negative coping is enhanced.

Regarding hypothesis 2, this study verified that burnout among nurses could impair individuals' interpersonal relationships and lead to depression. Research on medical students has demonstrated that burnout among medical students is closely related to interpersonal problems [27]. According to COR theory, individuals’ social competence tends to be impaired when they suffer from emotional exhaustion [46]. Thus, these individuals may experience more challenges in interacting in social situations and then engage in less-than-appropriate social behavior [13]. Furthermore, interpersonal relationships have been proven to be an important predictor of depression [47, 48]. The lack of social skills and poor speech can cause problems in interpersonal relationships, and subsequently, this interpersonal stress can lead to depression [49]. Regardless of the reasons, interpersonal relationship distress predicts individuals’ depression levels [50]. Therefore, the relationship between burnout and depression could be partially mediated by interpersonal relationships.

The results of this study indicated that the mediating effect of interpersonal relationships was significantly larger than that of positive coping styles, and this difference occurred mainly in the second half of the mediation model. Charles indicated that an individual's living environment is a complete ecosystem, including family, friends, work, etc. [51]. Human beings are the subject of continuous interaction with various ecosystems [51]. Therefore, we infer that when an individual's interpersonal relationships are impaired, this may lead to an imbalance in the system that causes psychological problems. As individuals adopt less positive coping styles, they may be more inclined to adopt avoidance, substance abuse, and other coping styles due to psychological defense mechanisms, which can somewhat buffer the onset of negative emotions [52–54]. However, whether the difference between the two mediating effects stems from the above mechanism needs to be further explored.

### Moderated analysis of being a frontline nurse for COVID-19 patients

The results of moderated analysis confirmed hypothesis 3, as burnout could affect depression among nurses directly, and the direct impact of burnout could be magnified among frontline nurses caring for COVID-19 patients. Compared to those who were nonfrontline nurses for COVID-19 patients, the association between burnout and depression increased significantly. Similar to the results of the moderated analysis of this study, a study focused on 987 subjects also stated that perceived health risk related to COVID-19 significantly affected the relationship between burnout and job performance [32]. A safe workplace serves as a work resource that can assist in reducing employee burnout and improving employee performance [55]. During the COVID-19 pandemic, a safe workplace could contribute to reducing employees' psychological problems by reducing their fear of external risks and increasing their sense of psychological security [55]. When nurses are exposed to high-risk environments for prolonged periods of time, such as being confronted with chronic uncertainty and fear regarding COVID-19, psychological security may be diminished, resulting in nurses being more vulnerable to mental health problems. Additionally, COVID-19 is a severe social concern that affects multiple aspects of society and is more than a medical issue [56]. In contrast to the common stressors that are confronted in daily life, COVID-19 is unpredictable, causing great uncertainty and fear among employees [56]. When employees are exposed to high levels of pressure, their psychological resilience may be lost and have a negative impact on their mental status [32]. Thus, frontline nurses for COVID-19 patients who suffer from burnout are more vulnerable to depression.

## Strengths and limitations

This study explored the possible mechanisms of burnout-induced depression, corroborated the potential mediating role of positive coping styles and interpersonal relationships and provided a theoretical basis for mitigating the negative effects of burnout on nurses' psychological status. In addition, this study explored the role of specific environmental factors in the relationship between burnout and depression, demonstrating that burnout is more likely to cause depression among frontline nurses for COVID-19 patients, suggesting that frontline nurses for COVID-19 patients may need more targeted psychological support.

This study has limitations. First, this was a cross-sectional study, and how depression is caused by burnout among nurses over time remains unclear. Further longitudinal studies are needed. Second, only the mediating role of the overall category of positive coping styles was investigated in this study, and specific coping styles were not included. Future studies that pay attention to specific coping styles, such as problem-centered coping, are needed. Third, this study only compared the difference between frontline and nonfrontline nurses for COVID-19 patients. Nurses at fever clinics and isolation wards are also frontline nurses for COVID-19 patients, while nurses of common internal medicine and surgery units, operating rooms, and emergency medicine facilities are nonfrontline nurses; however, these departments may differ from one another. Future studies should be precise about the differences between each department. Furthermore, due to geographical and human resource constraints, only hospitals that could contact the person in charge were selected for this study, rather than sampling evenly in each province of China, which is one of the limitations of this study. Lastly, this study excluded questionnaires that contained extreme values which might lead to loss of information. Better statistical methods are needed.

## Conclusion

In summary, this study investigated the relationship between burnout and depression and verified the mediating role of positive coping styles and interpersonal relationships. Furthermore, this study took environmental factors into consideration, and the direct association between burnout and depression could be magnified by being a frontline nurse for COVID-19 patients. Therefore, mental health education for nurses should be enhanced to promote effective coping with stress and improve interpersonal relationships among nurses. In addition, different mental health interventions should be implemented for different types of nurses, and nurses caring for diagnosed patients may need more mental health support.

## Data Availability

The datasets used and/or analyzed during the current study are available from the corresponding author on reasonable request.

## References

[1] Yoruk S, Guler D (2021). The relationship between psychological resilience, burnout, stress, and sociodemographic factors with depression in nurses and midwives during the COVID-19 pandemic: a cross-sectional study in Turkey. Perspect Psychiatr Care.

[2] Organization WH: Weekly epidemiological update on COVID-19 - 5 23 November 2022. In.; 2022.

[3] Lai J, Ma S, Wang Y, Cai Z, Hu J, Wei N, Wu J, Du H, Chen T, Li R (2020). Factors Associated With Mental Health Outcomes Among Health Care Workers Exposed to Coronavirus Disease 2019. Jama Network Open.

[4] de Pablo GS, Vaquerizo-Serrano J, Catalan A, Arango C, Moreno C, Ferre F, Shin JI, Sullivan S, Brondino N, Solmi M (2020). Impact of coronavirus syndromes on physical and mental health of health care workers: systematic review and meta-analysis. J Affect Disord.

[5] Tosun A, Tosun H, Ozkaya BO, Erdogan Z, Gul A: "Sleep Quality and Depression Level in Nurses in COVID-19 Pandemic". Omega-Journal of Death and Dying 2022. 10.1177/00302228221123159 PMC9424104 36036180

[6] Doo E-Y, Kim M, Lee S, Lee SY, Lee KY (2021). Influence of anxiety and resilience on depression among hospital nurses: a comparison of nurses working with confirmed and suspected patients in the COVID-19 and non-COVID-19 units. J Clin Nurs.

[7] Maslach C, Leiter MP (2016). Understanding the burnout experience: recent research and its implications for psychiatry. World Psychiatry.

[8] McManus IC, Winder BC, Gordon D (2002). The causal links between stress and burnout in a longitudinal study of UK doctors. Lancet.

[9] Brown SD, Goske MJ, Johnson CM (2009). Beyond substance abuse: stress, burnout, and depression as causes of physician impairment and disruptive behavior. Journal of the American College of Radiology : JACR.

[10] de Vasconcelos EM (2018). Figueiredo de Martino MM, de Souza Franca SP: burnout and depressive symptoms in intensive care nurses: relationship analysis. Rev Bras Enferm.

[11] Quintas S, Queiros C, Marques A, Orvalho V (2017). Nurses and their health at work: the relationship between depression and burnout. International Journal on Working Conditions.

[12] Tokur ME, Ergan B, Aydin K, Caliskan T, Savran Y, Yaka E, Koca U, Comert B, Gokmen N (2018). Depression and burnout frequency in nurses working in tertiary intensive care units. Journal of Medical and Surgical Intensive Care Medicine.

[13] Chen C, Meier ST: Burnout and depression in nurses - a systematic review and meta-analysis (vol 124, 104099, 2021). Int J Nurs Stud. 2022, 127. 10.1016/j.ijnurstu.2021.104099 34715576

[14] Leiter MP, Durup J (1994). The discriminant validity of burnout and depression - a confirmatory factor-analytic study. Anxiety Stress Coping.

[15] Stelnicki AM, Jamshidi L, Angehrn A, Hadjistavropoulos HD, Carleton RN (2021). Associations between burnout and mental disorder symptoms among nurses in Canada. Canadian Journal of Nursing Research.

[16] Happell B, Dwyer T, Reid-Searl K, Burke KJ, Caperchione CM, Gaskin CJ (2013). Nurses and stress: recognizing causes and seeking solutions. J Nurs Manag.

[17] Yoshizawa K, Sugawara N, Yasui-Furukori N, Danjo K, Furukori H, Sato Y, Tomita T, Fujii A, Nakagam T, Sasaki M (2016). Relationship between occupational stress and depression among psychiatric nurses in Japan. Arch Environ Occup Health.

[18] Folkman S, Lazarus RS, Gruen RJ, Delongis A (1986). Appraisal, coping, health-status, and psychological symptoms. J Pers Soc Psychol.

[19] Chen J, Li J, Cao B, Wang F, Luo L, Xu J (2020). Mediating effects of self-efficacy, coping, burnout, and social support between job stress and mental health among young Chinese nurses. J Adv Nurs.

[20] Garcia-Arroyo JA, Osca Segovia A: Work overload and emotional exhaustion in university teachers: moderating effects of coping styles. Universitas Psychologica 2019, 18(2).

[21] Pedro Martinez J, Mendez I, Ruiz-Esteban C, Fernandez-Sogorb A, Manuel Garcia-Fernandez J (2020). Profiles of Burnout, Coping Strategies and Depressive Symptomatology. Front Psychol.

[22] Shao R, He P, Ling B, Tan L, Xu L, Hou Y, Kong L, Yang Y (2020). Prevalence of depression and anxiety and correlations between depression, anxiety, family functioning, social support and coping styles among Chinese medical students. BMC Psychol.

[23] Bakker AB, Schaufeli WB, Sixma HJ, Bosveld W, Van Dierendonck D (2000). Patient demands, lack of reciprocity, and burnout: a five-year longitudinal study among general practitioners. J Organ Behav.

[24] Khanji MY, Maniero C, Ng S, Siddiqui I, Gupta J, Crosby L, Antoniou S, Khan R, Kapil V, Gupta A (2021). Early and Mid-Term Implications of the COVID-19 Pandemic on the Physical, Behavioral and Mental Health of Healthcare Professionals: The CoPE-HCP Study Protocol. Front Psychol.

[25] Yoon HS, Kim G-H, Kim J (2011). Effectiveness of an interpersonal relationship program on interpersonal relationships, self-esteem, and depression in nursing students. J Korean Acad Nurs.

[26] Zlotnick C, Kohn R, Keitner G, Della Grotta SA (2000). The relationship between quality of interpersonal relationships and major depressive disorder: findings from the National Comorbidity Survey. J Affect Disord.

[27] Bolatov AK, Seisembekov TZ, Smailova DS, Hosseini H (2022). Burnout syndrome among medical students in Kazakhstan. BMC Psychol.

[28] Puto G, Jurzec M, Leja-Szpak A, Bonior J, Muszalik M, Gniadek A (2022). Stress and Coping Strategies of Nurses Working with Patients Infected with and Not Infected with SARS-CoV-2 Virus. Int J Environ Res Public Health.

[29] Thobane KF, Mulaudzi FM, Moagi MM (2022). Improvement of the psychosocial support for frontline nurses in public hospitals during COVID-19 pandemic. J Nurs Manag.

[30] Li J, Su Q, Li X, Peng Y, Liu Y (2021). COVID-19 negatively impacts on psychological and somatic status in frontline nurses. J Affect Disord.

[31] Johns G: Advances in the Treatment of Context in Organizational Research. In: Annual Review of Organizational Psychology and Organizational Behavior, Vol 5. Volume 5, edn. Edited by Morgeson F; 2018: 21–46.

[32] Thinh-Van V, Vo-Thanh T, Chi H, Nguyen Phong N, Van Duy N, Zaman M (2022). The role of perceived workplace safety practices and mindfulness in maintaining calm in employees during times of crisis. Hum Resour Manage.

[33] Zakeri MA, Rahiminezhad E, Salehi F, Ganjeh H, Dehghan M (2021). Burnout, Anxiety, Stress, and Depression Among Iranian Nurses: Before and During the First Wave of the COVID-19 Pandemic. Front Psychol.

[34] Mao X, Lin X, Liu P, Zhang J, Deng W, Li Z, Hou T, Dong W (2023). Impact of Insomnia on burnout among chinese nurses under the regular COVID-19 epidemic prevention and control: parallel mediating effects of anxiety and depression. Int J Public Health.

[35] Kim HJ, Lee GH (2022). A comparative study of the psychological impacts of tasks related and unrelated to COVID-19 on nurses: a cross-sectional study. Journal of Yeungnam medical science.

[36] Antony MM, Bieling PJ, Cox BJ, Enns MW, Swinson RP (1998). Psychometric properties of the 42-item and 21-item versions of the depression anxiety stress scales in clinical groups and a community sample. Psychol Assess.

[37] Lovibond PF, Lovibond SH (1995). The structure of negative emotional states - comparison of the depression anxiety stress scales (DASS) with the beck depression and anxiety inventories. Behav Res Ther.

[38] Schutte N, Toppinen S, Kalimo R, Schaufeli W (2000). The factorial validity of the maslach burnout inventory-general Survey (MBI-GS) across occupational groups and nations. J Occup Organ Psychol.

[39] ChaoPing Li KS (2003). The impact of distributive and procedural fairness on job burnout. Acta Psychol Sin.

[40] Deng R (1999). Psychological diagnosis of college students.

[41] Xie Y (1998). A preliminary study of the reliability and validity of the simplified coping style questionnaire. Chin J Clin Psychol.

[42] Wang Y, Wang P (2019). Perceived stress and psychological distress among chinese physicians the mediating role of coping style. Medicine.

[43] Wang Y, Xiao H, Zhang X, Wang L (2020). The Role of Active Coping in the Relationship Between Learning Burnout and Sleep Quality Among College Students in China. Front Psychol.

[44] Zhou J, Yang Y, Qiu X, Yang X, Pan H, Ban B, Qiao Z, Wang L, Wang W (2016). Relationship between Anxiety and Burnout among Chinese Physicians: A Moderated Mediation Model. Plos One.

[45] Hobfoll SE (1989). Conservation of resources - a new attempt at conceptualizing stress. Am Psychol.

[46] Loh JMI, Saleh A: Lashing out: emotional exhaustion triggers retaliatory incivility in the workplace. Heliyon 2022, 8(1). 10.1016/j.heliyon.2021.e08694 PMC8749205 35036596

[47] Lee EJ (2014). 신승화: The Effect of Health Promotion Behavior on Fatigue and Depression among Shift Nurses. Journal of Korea Academia-Industrial cooperation Society.

[48] Sun BH (2020). hae kk, Lee D-G: the relationships among anger expression, interpersonal relationship, and depression in nursing students. Journal of Korea Academia-Industrial cooperation Society.

[49] Starr LR, Davila J (2008). Excessive reassurance seeking, depression, and interpersonal rejection: a meta-analytic review. J Abnorm Psychol.

[50] Eberhart NK, Hammen CL (2006). Interpersonal predictors of onset of depression during the transition to adulthood. Pers Relat.

[51] Fagan K (2017). Understanding human behavior and the social environment. Community Dev.

[52] Shin M, Kemps E (2020). Media multitasking as an avoidance coping strategy against emotionally negative stimuli. Anxiety Stress Coping.

[53] Fitzpatrick S, Khoury JE, Krantz L, Zeifman R, Kuo JR (2019). Next-day effects of dysfunctional and functional emotion regulation and the moderating role of experiential avoidance. J Contextual Behav Sci.

[54] Connolly RD, Noel V, Mezo PG (2017). Self-evaluation as a mediating variable between substance abuse and stress. Int J Ment Heal Addict.

[55] Lee H (2021). Changes in workplace practices during the COVID-19 pandemic: the roles of emotion, psychological safety and organisation support. Journal of Organizational Effectiveness-People and Performance.

[56] Trougakos JP, Chawla N, McCarthy JM (2020). Working in a pandemic: exploring the impact of COVID-19 health anxiety on work, family, and health outcomes. J Appl Psychol.

